# Changes in Health-Promoting Behaviors and Their Association with Weight Loss, Retention, and Engagement on a Digital Program: Prospective Study

**DOI:** 10.3390/nu14224812

**Published:** 2022-11-14

**Authors:** Heather Behr, Sydney Earl, Annabell Suh Ho, Jihye Lee, Ellen Siobhan Mitchell, Meaghan McCallum, Christine N. May, Andreas Michaelides

**Affiliations:** 1 Department of Integrative Health, Saybrook University, 55 W Eureka St., Pasadena, CA 91103, USA; 2 Department of Psychology, North Carolina State University, Poe Hall, 2310 Stinson Dr., Raleigh, NC 27695, USA; 3 Academic Research, Noom, 450 W 33rd St., New York, NY 10001, USA; 4 Moody College of Communication, The University of Texas at Austin, 300 W Dean Keeton St., Austin, TX 78712, USA

**Keywords:** mHealth, obesity, weight management, health-promoting behaviors

## Abstract

Health-promoting lifestyle behaviors (e.g., as measured by the HPLP-II) are associated with reductions in lifestyle disease mortality, as well as improved well-being, mental health, and quality of life. However, it is unclear how a weight-management program relates to a broad range of these behaviors (i.e., health responsibility, physical activity, nutrition, spiritual growth, interpersonal relations, and stress management), especially a fully digital program on which individuals have to self-manage their own behaviors in their daily lives (with assistance from a virtual human coach). In the context of a digital setting, this study examined the changes in health-promoting behaviors over 12 months, as well as the associations between health-promoting behaviors and weight loss, retention, and engagement, among participants who self-enrolled in a mobile CBT-based nutritionally focused behavior change weight management program (*n* = 242). Participants lost a statistically significant amount of weight (M = 6.7 kg; SD = 12.7 kg; t(80) = 9.26, *p* < 0.001) and reported significantly improved overall health-promoting lifestyle behaviors (i.e., HPLP-II summary scores), as well as, specifically, health responsibility, physical activity, nutrition, spiritual growth, stress management, and interpersonal relations behaviors from baseline to 6 months and from 6 months to 12 months (all *p*s < 0.008). Health-promoting behaviors at 6 months (i.e., learned health-promoting behaviors) compared to baseline were better predictors of retention and program engagement. A fully digital, mobile weight management intervention can improve HPLP-II scores, which, in turn, has implications for improved retention, program engagement, and better understanding the comprehensive effects of weight management programs, particularly in a digital setting.

## 1. Introduction

Lifestyle diseases, or non-communicable diseases, account for the world’s leading causes of death, including heart disease, stroke, cancer, chronic respiratory diseases, and Type 2 diabetes [1,2]. These diseases are greatly influenced by health-related (or health-promoting) behaviors. Despite the critical role these behaviors play in promoting health, levels of individual health-promoting behaviors are generally low across the globe [3]. Health-promoting behaviors encompass several different dimensions of health including health responsibility, physical activity, nutrition, spiritual growth, interpersonal relations, and stress management [4]. Many studies have assessed various dimensions of health-promoting behaviors, finding that these behaviors not only play a key role in addressing and preventing both communicable and non-communicable diseases, but also positively influence individual well-being, mental health, and overall quality of life [5,6,7,8,9,10,11]. 

There is reason to believe that weight management programs may impact the broad range of health-promoting behaviors [12,13]. Reviews suggest that participation in programs focused on health-promoting behaviors is often associated with significant improvements in nutrition and physical activity-related behaviors, as well as weight-related outcomes [14,15]. However, there are few studies that comprehensively examine the potential impact of weight management interventions on improving all the dimensions of health-promoting behaviors, including spiritual growth (e.g., exposure to new experiences and challenges), interpersonal relations, stress management, nutrition, physical activity, and health responsibility (e.g., read or watch TV programs about improving health), each of which has been individually shown to relate to important health outcomes [16,17,18,19]. Studies that have assessed health-related interventions in specific populations (i.e., women with multiple sclerosis; nurses) have found success in improving all or many dimensions of health-promoting behaviors [20,21]. We aimed to add to the literature by assessing the same outcomes prospectively in a broader population of adults with obesity or overweight in a behavior change weight-management program. Additionally, studies that have comprehensively assessed health-promoting behaviors have found that many, if not all, dimensions of health-promoting behaviors, are related to quality of life, self-efficacy, and BMI in specific populations [20,21,22,23]. Because the facets of health-promoting behaviors are associated with better overall health, well-being, and weight-loss related outcomes, it is important to examine how they relate to participating in a behavior change weight-management program. This is especially the case in a fully digital setting, in which individuals self-manage their participation as well as their behavioral changes without factors common to in-person or clinical settings such as frequent contact from researchers or staff and attendance checks. In this study, we accordingly assess health-promoting behaviors in the context of a fully digital behavior change weight-management program.

Furthermore, there is reason to believe that programs that incorporate nutrition curriculum and cognitive behavioral therapy (CBT) techniques could lead to improved health-promoting behaviors, in many if not all dimensions. More specifically, gaining nutrition knowledge (e.g., learning to understand food labels, select healthier foods, or identify healthy portion sizes) is associated with better diet quality [24,25]. While there is limited research examining the direct relationship of CBT influence on increasing health-promoting behaviors [26], CBT techniques are linked to outcomes such as improved physical functioning, physical health, and stress management [27,28,29]. The question still remains as to whether CBT techniques could improve health-promoting behaviors in the context of a weight management program. Another gap in current literature relates to the relationship between health-promoting behaviors and program engagement, retention, and weight loss. There is reason to believe that an increase in health-promoting behaviors may be related to program engagement and weight loss [12,30]. However, it is unclear how both baseline and changes in health-promoting behaviors over time relate to program engagement and weight loss. 

Therefore, the aims of this study were to explore changes in health-promoting lifestyle behaviors (HPLP-II) (i.e., baseline, 6-month, and 12-month scores), as well as the associations between baseline and 6-month health-promoting behaviors and 12-month weight loss, engagement, and retention in a mobile behavior change weight-management program that includes nutrition curriculum and CBT techniques (Noom Weight; NW). To evaluate such behaviors, we utilize the Health-Promoting Lifestyle Profile II (HPLP-II), a common and valid measure encompassing the variety of health-promoting behaviors described above [31]. Exploring these relationships can assess how this type of weight-loss program relates to health-related outcomes beyond weight loss, namely a broader range of health-promoting behaviors which have not been evaluated on a fully digital weight management program before. In addition, exploring the relationship between health-promoting behaviors and weight loss, engagement, and retention can inform targeting at-risk individuals (using baseline associations) and whether improvement in health-promoting behaviors at 6 months better predicts 12-month outcomes than baseline. 

## 2. Materials and Methods

### 2.1. Procedure

This study was a single-arm prospective cohort design in which a random subset of Noom Weight subscribers who signed up for the program between November and December 2020 were invited to participate in the study. Participants were invited to complete the baseline questionnaire in which they were given the opportunity to consent. Participants also consented to the use of their de-identified program data for research purposes. Those who completed the baseline questionnaire were invited to participate in follow-up surveys at 6 and 12 months regardless of whether they were still using the program. Program engagement (e.g., reading articles, logging dietary intake, etc.) was directly recorded within the mobile application throughout the 12 months. To measure real-world engagement, participants were not given specific minimum engagement requirements. Participants who completed the study were compensated for their participation. At baseline, participants were given a free 4-month subscription to Noom Weight, as well as USD 30 for completing the survey at 6 months and USD 30 for completing the survey at 12 months. Because participants had already signed up for an 8-month subscription, compensation ensured that participants had access to Noom for a total of 12 months. All procedures were approved by the Advarra Institutional Review Board.

### 2.2. Participants

Participants included adults who voluntarily signed up for a behavior change weight-management program (Noom Weight; NW) between the months of November and December 2020. A random subset of 3000 users who elected to subscribe to NW for 8 months following a two-week trial period were invited to participate in this prospective study. Inclusion criteria assessed at signup were: located within the US, BMI of 25 or higher, and English speaking.

### 2.3. Intervention

Noom Weight (NW) is a digital weight management program that has been shown to be effective in promoting clinically significant weight loss and behavioral change improvements through behavior change techniques [32]. NW provides users with self-monitoring features for food, exercise, and weight monitoring, as well as access to a virtual 1:1 human coach, a support group facilitated by a human coach, and a daily curriculum that includes psychoeducation surrounding nutrition, physical activity, and sustainable behavioral change. While there are no requirements for program engagement, individuals are encouraged to utilize these self-monitoring features as consistently as possible (e.g., daily or weekly) as well as read the assigned daily articles that take 5–10 min in total to read. NW’s approach is informed by acceptance and commitment therapy (ACT), dialectical behavior therapy (DBT), and cognitive behavioral therapy (CBT), all of which aid in behavior change and weight control [33,34,35]. Components of these approaches are incorporated into asynchronous 1:1 coaching, as well as NW’s curriculum. For example, interactive, daily articles introduce the framework (e.g., what is CBT), describe its components (e.g., what are cognitive distortions), and provide practical tips and applicable examples for users to incorporate into their life (e.g., step-by-step identification and reappraisal of a participant’s cognitive distortion). Previous work on NW has shown that engagement with this type of curriculum is associated with increased self-compassion over time [36]. Importantly, the literature also suggests a strong relationship between self-compassion and several dimensions of health-promoting behaviors [37,38]. NW’s curriculum also encourages other dimensions of health-promoting behaviors by providing relevant information regarding health responsibility (e.g., paying attention to one’s sleep), physical activity (e.g., ideas for integrating exercise into one’s daily life), spiritual growth (e.g., meditation practices), interpersonal relations (e.g., tips for practicing skills with partners or friends), and stress management (e.g., examples of coping skills). 

Additionally, NW incorporates many features which aim to encourage a specific dimension of health-promoting behaviors—proper nutrition. Namely, the curriculum provides education on healthy eating and nutrition informed by empirical research as well as MyPlate recommendations in order to enable a sustainable, high-quality diet [39,40]. In conjunction with the curriculum, users are encouraged to log their food according to a color system that categorizes foods based on energy density, in terms of high (formerly red, now orange), medium (yellow), and low (green) energy density. NW users are provided with information on the foods they log, including portion size, calories, caloric density, and other nutritional information. Previous work has shown that adherence to NW’s color system is associated with greater weight loss [41]. Previous work describes NW in greater detail [36,42].

### 2.4. Measures

The primary measure was health-promoting behaviors while secondary measures included weight loss, engagement, and retention. Identical surveys were completed at baseline and the 6 and 12 months follow-up periods, with the addition of demographic information assessed at baseline. 

Health-promoting behaviors were measured with a survey at baseline, 6 and 12 months using the Health-Promoting Lifestyle Profile II (HPLP-II) questionnaire [4]. The HPLP-II has been widely used across several linguistic and cultural groups in many studies, each of which has found the HPLP-II to be valid and reliable [43,44,45]. This 52-item scale yields a total score, as well as subscale scores on six dimensions, encompassing behaviors related to health responsibility, physical activity, nutrition, spiritual growth, interpersonal relations, and stress management. For example, nutritional health-promoting behaviors include “Eat 2-4 servings of fruit each day” and “Read labels to identify nutrients, fats, and sodium content in packaged food”. The HPLP-II summary score was calculated by averaging all the responses given for the 52 items. To assess each dimension of the HPLP-II (e.g., health responsibility, physical activity, etc.), items corresponding to each dimension were averaged accordingly. Response scales ranging from 1 (never) to 4 (routinely) and higher scores indicate more frequent health-promoting behaviors. Internal reliabilities ranged from α = 0.91 to 0.93 for baseline and follow-up at 6 and 12 months, indicating excellent reliability.

Weight loss was calculated by subtracting the most recent weight from the initial weight logged (i.e., self-reported) within the program. When in-app logged weigh-ins were not available, self-reported weight data (from the 12-month survey) were supplemented for 12-month weight loss. 

Engagement was extracted from the program database. These measures encompassed the ways in which individuals were encouraged to actively participate in the program. More specifically, the program measured the total frequency of: (1) meals logged; (2) weigh-ins completed; (3) exercises logged; (4) messages sent to individual coaches; (5) articles read; (6) group activities (posts, post hearts, thread posts, thread post hearts); and (7) steps per week. These engagement behaviors have been shown to be individually associated with weight loss in previous studies [32]. To create a total engagement score, these seven frequencies were normalized and summed, following previous work [10,32]. Each dimension of the engagement measure (e.g., meals logged, weigh-ins, exercises logged, messages sent to coaches, articles read, group activities, and steps) separately showed the same pattern of cumulative engagement score findings.

Retention was measured using engagement data extracted from the program database. A participant with any engagement activity with the program for the month that retention was considered retained; a participant with no program engagement was considered not retained.

### 2.5. Statistical Analysis

Linear mixed effects models were used since they are robust models for repeated-measures analysis [46]. To examine how health-promoting behaviors changed over time, linear mixed effects models predicted HPLP-II scores with a time point (6 months and 12 months) as a fixed effect and each participant as a random effect. Baseline was used as the reference value for the time point, meaning that 6-month and 12-month scores were compared to the baseline scores. To assess whether the baseline (or 6-month) HPLP scores predicted outcomes (i.e., weight loss, retention, and engagement) at 12 months, linear mixed effects models were conducted, predicting the outcome using the baseline (or 6-month) HPLP score, age, gender, and initial BMI as fixed effects. Separate univariate models were run for each HPLP score (e.g., nutrition, physical activity). Analyses were conducted in R version 1.4.1106 with a 2-tailed alpha of 0.05.

## 3. Results

### 3.1. Baseline Characteristics

A total of 691 participants enrolled in the current study and completed a baseline survey. Of these participants, 394 also completed a follow-up survey at 6 months and 242 completed a follow-up survey at 12 months, with a survey dropout rate of 57% at 6 months and 61% at 12 months. The participants who completed both baseline and subsequent surveys did not significantly differ in any demographic variable from the individuals who only completed the baseline survey. Participants were mainly female (n = 511, 74%). The average age was 44.04 (SD = 12.42) and the average BMI at baseline was 40.17 (SD = 5.42). At 6 months, the participants lost an average of 6.3% of their initial weight (SD = 6.3%; M = 7.4 kg; SD =7.4 kg), with 54.1% achieving weight loss ≥ 5% of their initial body weight. At 12 months, participants lost an average of 5.6% of their initial weight (SD = 10.8%; M = 6.7 kg; SD =12.7 kg), with 41.7% achieving weight loss ≥ 5% of their initial body weight. Weight significantly decreased from baseline to 6- and 12-month follow-up (*p*s < 0.001).

### 3.2. Changes in HPLP-II Scores

First, we examined the difference in HPLP-II scores between baseline and 6 months (Table 1). The summary HPLP-II score, as well as most subscale scores, improved over the 6-month period. More specifically, the HPLP-II summary score (a combined score of all health-promoting behaviors) significantly improved from baseline to 6 months (t(482) = 5.81, β = 0.11, SE = 0.02, *p* < 0.001). Similarly, health responsibility behaviors significantly improved over the course of the program (t(482) = 3.25, β = 0.09, SE = 0.03, *p* =0.001), as well as physical activity (t(482) = 8.28, β = 0.26, SE = 0.03, *p* < 0.001), nutrition (t(482) = 6.78, β = 0.16, SE = 0.02, *p* < 0.001), spiritual growth (t(482) = 2.77, β = 0.08, SE = 0.03, *p* < 0.001), and stress management behaviors (t(482) = 2.54, β = 0.07, SE = 0.07, *p* = 0.01). There was no significant change in the dimension of interpersonal relations behaviors (t(482) = 0.43, β = 0.01, SE = 0.02, *p* = 0.67). In other words, the overall HPLP-II score significantly improved, as did the health responsibility, physical activity, nutrition, spiritual growth, and stress management behaviors from baseline to 6 months; interpersonal relations behaviors did not change from baseline to 6 months.

We then examined the difference in HPLP-II scores between baseline and 12 months (Table 1). The summary HPLP-II score, as well as all subscale scores, improved over the 12-month period. More specifically, the HPLP-II summary score significantly improved from baseline to 12 months (t(482) = 8.00, β = 0.15, SE = 0.02, *p* < 0.001). Similarly, health responsibility behaviors significantly improved over the course of the program (t(482) = 7.26, β = 0.19, SE = 0.03, *p* < 0.001), as well as with physical activity (t(482) = 7.33, β = 0.23, SE = 0.03, *p* < 0.001), nutrition (t(482) = 5.05, β = 0.12, SE = 0.02, *p* < 0.001), spiritual growth behaviors (t(482) = 5.23, β = 0.15, SE = 0.03, *p* <.001), stress management (t(482) = 5.40, β = 0.15, SE = 0.03, *p* < 0.001), and interpersonal relations behaviors (t(482) = 2.65, β = 0.07, SE = 0.02, *p* = 0.008). In other words, the overall HPLP-II score significantly improved, as well as all other scores (health responsibility, physical activity, nutrition, spiritual growth, interpersonal relations, and stress management behaviors) from baseline to 12 months.

### 3.3. HPLP-II and Weight Loss

Then, we examined whether baseline HPLP-II scores were associated with the amount of weight participants lost at 12 months (Table 2). When controlling for relevant variables (i.e., age, gender, and baseline BMI) in a series of multiple regressions, only the baseline nutrition score was a significant predictor of weight loss at 12 months (B = −4.32, SE = 1.84, *p* = 0.02). 

We then examined whether the 6-month HPLP-II scores were associated with weight loss at 12 months. When controlling for relevant variables (i.e., age, gender, and baseline BMI) in a series of multiple regressions, only nutrition behaviors at 6 months were significantly related to weight loss at 12 months (B = −4.23, SE = 1.78, *p* = 0.02). Physical activity behavior (B = −2.30, SE = 1.25, *p* = 0.07) was also a marginally significant predictor of 12-month weight loss. No other HPLP-II scores were significantly associated with 12-month weight loss. In other words, higher nutrition behavior scores at baseline and 6 months were significantly associated with weight loss at 12 months.

### 3.4. HPLP-II and Engagement

Finally, we examined whether baseline HPLP-II scores were associated with engagement at 12 months (Table 2). When controlling for relevant variables (i.e., age, gender, and baseline BMI) in a series of mixed effect models, higher baseline HPLP-II summary scores were significantly associated with overall higher program engagement at 12 months (t(287.67) = 3.71, B = 2.02, SE = 0.54, *p* < 0.001). Additionally, higher baseline scores of physical activity (t(294.44) = 5.84, B = 1.96, SE = 0.34, *p* < 0.001), nutrition (t(297.08) = 4.26, B = 2.23, SE = 0.52, *p* < 0.001, and spiritual growth behaviors (t(299.90) = 2.59, B = 0.91, SE = 0.35, *p* = 0.001) were associated with higher program engagement at 12 months. Stress management behavior (t(298.79) = 1.70, B = 0.67, SE = 0.40, *p* = 0.09) was also a marginally significant predictor for engagement. No other baseline HPLL-II scores were significantly associated with engagement at 12 months. In other words, baseline HPLP-II summary scores as well as physical activity, nutrition, and spiritual growth behavior scores at baseline were significantly associated with engagement at 12 months. Stress management behavior scores at baseline were associated with engagement at 12 months with marginal significance.

We then examined whether 6-month HPLP-II scores were associated with engagement at 12 months. When controlling for relevant variables (i.e., age, gender, and baseline BMI) in a series of mixed-effect models, higher 6-month HPLP-II summary scores were associated with overall higher program engagement at 12 months (t(387.15) = 5.46, B = 2.66, SE = 0.49, *p* < 0.001). Additionally, higher scores of physical activity (t(438.25) = 7.54, B = 1.94, SE = 0.26, *p* < 0.001), nutrition (t(389.58) = 5.72, B = 2.39, SE = 0.42, *p* < 0.001), spiritual growth (t(399.96) = 3.95, B = 1.33, SE = 0.34, *p* < 0.001), and stress management behaviors (t(516.92) = 2.87, B = 1.11, SE = 0.39, *p* = 0.004) at 6 months were associated with higher program engagement at 12 months. No other 6-month HPLL-II scores were significantly associated with engagement at 12 months. In other words, 6-month HPLP-II summary scores as well as physical activity, nutrition, spiritual growth, and stress management behavior scores were significantly associated with engagement at 12 months.

### 3.5. HPLP-II and Retention

Then, we examined whether baseline HPLP-II scores were associated with retention at 12 months (Table 2). When controlling for relevant variables (i.e., age, gender, and baseline BMI) in a series of logistic regressions, higher baseline nutrition behaviors (OR = 2.28, B = 0.82, SE = 0.25, *p* = 0.001) were significantly associated with retention at 12 months. In other words, the higher the nutrition behavior score was at baseline, the more likely they were to stay with NW for 12 months. No other HPLL-II scores were significantly associated with retention at 12 months. 

When controlling for relevant variables (i.e., age, gender, and baseline BMI) in a series of logistic regressions, 6-month HPLP-II summary scores (OR = 2.11, B = 0.75, SE = 0.29, *p* = 0.009), as well as physical activity (OR = 1.80, B = 0.59, SE = 0.18, *p* < 0.001) and nutrition behaviors (OR = 3.01, B = 1.10, SE = 0.26, *p* < 0.001) were associated with retention at 12 months. In other words, the higher the physical activity, nutrition, and HPLP-II summary scores were at 6 months, the more likely they were to stay with NW for 12 months. No other HPLP-II scores were significantly associated with retention at 12 months.

## 4. Discussion

In this single-arm prospective study, we sought to examine changes in health-promoting lifestyle behaviors, as well as weight loss, engagement, and retention among individuals who had self-enrolled in a mobile behavior change weight-management program over the course of 12 months. We also observed how health-promoting behaviors at baseline and at 6 months (i.e., learned health-promoting behaviors) were associated with changes in weight loss, engagement, and retention. From baseline to 6 months, overall health-promoting behaviors (i.e., HPLP-II summary scores), as well as the dimensions of health responsibility, physical activity, nutrition, spiritual growth, and stress management behaviors significantly improved, while interpersonal relations behaviors did not. From baseline to 12 months, all of these behaviors significantly improved. Average weight loss from baseline to 12 months was also statistically significant. A greater number of health-promoting behaviors at 6 months (i.e., learned health-promoting behaviors) were significant predictors of program engagement and retention, compared to behaviors at baseline. 

Weight management programs and subsequent studies tend to focus on weight loss as the main outcome; however, our study is one of the first to highlight the breadth of benefits that can result from a behavior change weight-management program—namely, cultivating all health-promoting behaviors, particularly in a fully digital setting, which can differ from in-person or clinical ones which are more commonly studied. Health-promoting behaviors measured by the HPLP-II encapsulate a lifestyle that is a “multidimensional pattern of self-initiated actions and perceptions that serve to maintain or enhance the level of wellness, self-actualization, and fulfillment of the individual” [31]. These lifestyle factors have been established as pivotal in affecting health, such as a lower rate of disease and death [47,48] and greater quality of life [49,50]. Despite evidence of the beneficial influence of health-promoting behaviors and the increased prevalence of weight management programs, there are limited studies comprehensively examining these behaviors as outcomes, particularly on a digital program [51]. We extended this knowledge by showing that, in the context of NW, a mobile program incorporating nutrition curriculum and CBT techniques, nearly every dimension of health-promoting behaviors improved. These results corroborate studies that have explored the effects of a weight management program on one or more of these health-promoting behaviors. For example, research has shown that participation in behavior change weight-management programs to be related to increased health responsibility, nutrition, and physical activity behaviors [15,52,53]. There are no studies to our knowledge that explicitly evaluate spiritual growth (e.g., feeling that I am growing and changing in positive ways) as an improved outcome of a weight management program, though research in the context of weight loss has found similar variables, such as vitality (e.g., subjective feeling of enthusiasm and spirit) and self-compassion, to improve in similar programs [10,36,54]. Some health behaviors, including interpersonal relations and stress management, have been shown to influence weight loss [55,56,57]; however, this study is one of the first to show that these behaviors may improve in a weight management program. These results highlight the potential important role that this type of weight-management program (i.e., CBT-based, nutritionally focused) may play in influencing lifestyle changes related to weight loss and health-related outcomes, which should be confirmed in future work. Given these findings, it may be informative to further examine the causal relationship between weight-management programs and health-promoting behaviors, and in turn, their influence on sustained weight loss and long-term health outcomes.

Our results suggest that health-promoting behaviors, as measured by the HPLP-II, are related to weight loss, as well as program engagement and retention. For weight loss, nutrition behaviors were important at baseline, just as they are at 6 months, in predicting 12-month weight loss. Physical activity behaviors at 6 months were marginally associated with 12-month weight loss while baseline behaviors were not, suggesting a small potential learning effect with physical activity, but not for nutrition behaviors. While the relationship between nutritional behaviors (e.g., eating more fruits and vegetables or reading nutritional labels) and successful weight loss is well established [41,58,59,60,61], our results contribute to this literature by showing that nutritional behaviors are the most predictive at baseline out of all health-promoting behaviors. The nutritional behaviors measured in this study capture both dietary self-monitoring (e.g., measuring quantity of food eaten) and health literacy (e.g., interpreting food labels), which have been shown to be significant predictors of weight loss and maintenance [62,63,64]. Given that the associations uncovered in this study indicate that both baseline and learned nutrition behaviors may be important, NW may both be especially useful to those with existing healthy nutritional behaviors but also serve as a tool to improve nutritional behaviors through its food logging system as well as its curriculum-addressing topics such as health literacy (e.g., articles addressing what components to look for in a nutrition label) and diet (e.g., articles addressing the importance of eating fruits and vegetables). In the long term, providing individuals with tools and information that strengthen their confidence in their ability to make nutritional behavioral changes may also increase individuals’ self-efficacy, which has been linked to greater weight loss and long-term behavior change [65,66,67]. More specifically, preliminary research among women with abdominal obesity enrolled in a heart and weight management program highlighted that increases in HPLP-II scores (i.e., health-promoting behaviors) and initial weight loss was significantly correlated with diet self-efficacy [68]. Based on our results and the literature, future research should extend these findings by delineating the relationship between weight loss, diet self-efficacy, and health-promoting behaviors (i.e., specifically nutritional behaviors). 

Our results suggest that health-promoting behaviors at 6 months (i.e., learned health-promoting behaviors) were generally better predictors of program engagement and retention than baseline health-promoting behaviors. For retention, there was one significant predictor at baseline in comparison to three at 6 months (i.e., summary scores, physical activity, and nutrition). In regard to engagement, there were four significant HPLP-II predictors at baseline in comparison to five at 6 months (i.e., summary scores, physical activity, nutrition, spiritual growth, and stress management). This raises the possibility that learned behaviors (i.e., health-promoting behaviors learned or improved by the program) are more influential in predicting future retention and engagement in this setting. Future studies should investigate potential mediational pathways at work. For example, while few studies have explored the relationship between spiritual growth and program engagement and retention, research has shown a significant relationship between vitality (similar to spiritual growth) and adherence to behavioral recommendations [69]. Considering previous work, spiritual growth may also be related to engagement and retention as a result of increased resiliency (i.e., the ability to adjust to adversity). In several studies among nurses, resiliency was positively and significantly correlated with health-promoting behaviors [70,71,72,73]. In one of these studies, researchers found that, in addition to health-promoting behaviors, spiritual health (e.g., I think about how my life can be more fulfilling) was a significant predictor of increased resiliency [70]. Resiliency in the context of a weight-management program may influence an individual’s ability to maintain focus and a positive outlook. Given these studies and our results, it is possible that the HPLP-II dimension of spiritual growth (e.g., exposure to new experiences/challenges) may be helpful for active participation in a program either directly or as a result of increased resiliency. These findings call for a more in-depth assessment of the relationship between resiliency and health-promoting behaviors, including spiritual growth, among the broader population, as well as exploring the role resiliency may play in long-term weight-loss and behavioral change in individuals’ engagement and retention in a weight-management program. 

Studies in the context of a weight-management program have found that higher stress levels are related to a decreased likelihood to complete or engage with the program [74,75]. Our results on 6-month stress management behaviors predicting 12 month engagement build on this knowledge, suggesting that participants that develop stress management behaviors (e.g., use specific methods to control my stress) may be able counteract the effects of stress and, in turn, be more likely to engage in or complete a weight-management program. Further studies should tease apart this relationship.

Beyond engagement and retention at 12 months, the literature suggests that once individuals have developed these health-promoting behaviors, they are more likely to sustain weight loss, and habit changes in the long term, implying the potential long-lasting effects of their participation in a weight management program [32,47,76,77]. While our results contribute evidence for the relationship between health-promoting behaviors, weight loss, engagement, and retention, exploring the sustainability of this relationship will be important in informing long-term results. 

Limitations. This study explored behaviors in a real-world setting on a publicly available program. Because of this, a convenience sample of individuals that were self-enrolled in NW was used. Thus, no control group was used and findings may not generalize to populations who choose not to participate in a weight management program. Furthermore, while the participants who completed both baseline and subsequent surveys did not significantly differ in any demographic variable from the individuals who only completed the baseline survey, the rate of survey dropout in this study (57% at 6 months and 61% at 12 months) was relatively high, which tends to be common for online academic studies of mobile weight management programs [78,79]. As digital weight management programs continue to be implemented and assessed, future studies should measure in detail the reasons for attrition in digital studies. Participants also self-reported their own weight on the platform which was used to calculate the weight loss over time; however, participants were encouraged to weigh in at the same time each day as well as use the same scale throughout the program to keep the measurements of their weight consistent. Similar studies may increase the validity and reliability of weight by utilizing objective measurements of weight such as digital scales. Furthermore, the results may not generalize to all individuals who seek to lose weight, particularly given the high baseline BMI, which could be due to this sample’s desire for a longer plan (8-month program). Additionally, this study does not account for external environmental factors that may affect HPLP-II scores, such as education, mental health status, and sociodemographic factors [23,80,81]. Taking into account these external factors, some researchers suggest a more tailored or comprehensive approach to promoting health-related behaviors and outcomes. Future research can explore these factors, as well as psychological factors including self-efficacy and resiliency, and how they relate in the context of a weight management program. Finally, to validate and expand on the present study, research should explore the similar effects of health-promoting behaviors on weight loss, engagement, retention and long-term health outcomes, as well as intervention follow-up, over a time period longer than 12 months to best understand the long-term implications.

## 5. Conclusions

This study offers unique insight into how health-promoting behaviors change over time in a mobile CBT-based, nutritionally focused weight management program as well as the relationship between health-promoting behaviors and weight loss, engagement, and retention. 

Individuals who self-enrolled in the weight management program experienced improvements in all dimensions of health-promoting behaviors (e.g., stress management, health responsibility) over time. Given previous work, this could be because a CBT-based and nutritionally focused curriculum can help individuals cultivate overall health-promoting behaviors and nutrition-related knowledge, possibly through a combination of self-monitoring one’s diet, exercise, and weight, while learning the psychological concepts governing these behaviors. Investigating this relationship with randomized trials may provide further understanding regarding the most influential components of the program. 

Nutrition behaviors at baseline and 6 months were equally predictive of weight loss, highlighting the importance of nutritional behaviors in long-term weight loss. Although it is clear from previous work that nutritional behaviors are associated with weight loss, our results underscore that both baseline and learned nutritional behaviors may be useful to target individuals at risk of suboptimal weight loss. These findings can be extended by investigating other factors that may relate to the relationship between nutritional behaviors and long-term weight loss, such as self-efficacy [56]. Understanding this relationship may provide the basis for targeting specific behaviors and characteristics in order to most effectively promote weight change.

More 6-month health-promoting behaviors were predictive of outcomes such as engagement and retention than baseline behaviors, raising the possibility that learned behaviors are more influential than individuals’ baseline behaviors. These findings have implications for the importance of teaching health-promoting behaviors on the program and how programs may target at-risk individuals with health-promoting behavioral content and curricula in order to increase engagement and retention. Future research should evaluate to what extent these health-promoting behaviors relate to better long-term sustained health, quality of life, and weight maintenance in adults with obesity/overweight after using this type of program. Additionally, studies should utilize randomized controlled trials, as well as studies assessing factors related to retention in this context in order to more comprehensively understand these learned behavioral effects on health-related outcomes.

## Figures and Tables

**Table 1:** Health-Promoting Lifestyle Behaviors (HPLP-II) Overtime.

Outcome	Timeline	Mean Difference (i.e., Month 6–Baseline; Month 12–Baseline)	Test Statistic	*p*-Value	SE
HPLP-II Summary Score	Baseline–Month 6	+0.11	t(482) = 5.81	<0.001 ***	0.02
	Baseline–Month 12	+0.15	t(482) = 8.00	<0.001 ***	0.02
Health Responsibility	Baseline–Month 6	+0.09	t(482) = 3.25	0.001 ***	0.03
	Baseline–Month 12	+0.20	t(482) = 7.26	<0.001 ***	0.03
Physical Activity	Baseline–Month 6	+0.26	t(482) = 8.28	<0.001 ***	0.03
	Baseline–Month 12	+0.23	t(482) = 7.33	<0.001 ***	0.03
Nutrition	Baseline–Month 6	+0.15	t(482) = 6.78	<0.001 ***	0.02
	Baseline–Month 12	+0.11	t(482) = 5.05	<0.001 ***	0.02
Spiritual Growth	Baseline–Month 6	+0.08	t(482) = 2.77	0.006 **	0.03
	Baseline–Month 12	+0.16	t(482) = 5.23	<0.001 ***	0.03
Interpersonal Relations	Baseline–Month 6	+0.01	t(482) = 0.43	0.670	0.02
	Baseline–Month 12	+0.07	t(482) = 2.65	0.008 **	0.02
Stress Management	Baseline–Month 6	+0.08	t(482) = 2.54	0.011 *	0.03
	Baseline–Month 12	+0.16	t(482) = 5.40	<0.001 ***	0.03

Note: The mean difference (Baseline–Month 6; Baseline–Month 12) shows the difference overtime in each outcome (e.g., HPLP-II summary score, health responsibility, physical activity, etc.). Health responsibility behaviors, physical activity behaviors, nutrition behaviors, spiritual growth behaviors, interpersonal relations behaviors, and stress management behaviors are subscales of the HPLP-II scale. The HPLP-II summary score is an average of these subscales. The HPLP-II scale ranges from 1 to 4 and is coded such that higher scores denote more frequent health-promoting behaviors. *** *p* < 0.001, ** *p* < 0.01, * *p* < 0.05.

**Table 2:** HPLP-II Scores and 12 Month Weight Loss, Engagement, and Retention.

	12 Month Weight Loss		12 Month Engagement		12 Month Retention	
Predictor	Association between Baseline Score and 12-Month Weight Loss	Association between 6-Month Score and 12-Month Weight Loss	Association between Baseline Score and 12-Month Engagement	Association between 6-Month Score and 12-Month Engagement	Association between Baseline Score and 12-Month Retention	Association between 6-Month Score and 12-Month Retention
HPLP Summary Score	B = −0.77,	B = −2.97,	B = 2.02,	B = 2.66,	B = 0.38,	B = 0.75,
	s.e. = 1.91,	s.e. = 2.08,	s.e. = 0.54,	s.e. = 0.49,	s.e. = 0.26,	s.e. = 0.29,
	*p* = 0.70	*p* = 0.15	*p* < 0.001 ***	*p* < 0.001 ***	*p* = 0.14	*p* = 0.009 **
Health Responsibility Behaviors	B = 1.05,	B = 0.66,	B = 0.60,	B = 0.48,	B = 0.26,	B = 0.26,
	s.e. = 1.23,	s.e. = 1.66,	s.e. = 0.37,	s.e. = 0.36,	s.e. = 0.18,	s.e. = 0.22,
	*p* = 0.42	*p* = 0.69	*p* = 0.11	*p* = 0.19	*p* = 0.14	*p* = 0.24
Physical Activity Behaviors	B = 1.00,	B = −2.30,	B = 1.96,	B = 1.94,	B = 0.13,	B = 0.59,
	s.e. = 1.23,	s.e. = 1.25,	s.e. = 0.34,	s.e. = 0.26,	s.e. = 0.16,	s.e. = 0.18,
	*p* = 0.42	*p* = 0.07+	*p* < 0.001 ***	*p* < 0.001 ***	*p* = 0.42	*p* <.001 ***
Nutrition Behaviors	B = −4.32,	B = −4.23,	B = 2.23,	B = 2.39,	B = 0.82,	B = 1.10,
	s.e. = 1.84,	s.e. = 1.78,	s.e. = 0.52,	s.e. = 0.42,	s.e. = 0.25,	s.e. = 0.26,
	*p* = 0.02 *	*p* = 0.02 *	*p* < 0.001 ***	*p* < 0.001 ***	*p* <.001 ***	*p* < 0.001 ***
Spiritual Growth Behaviors	B = −0.54,	B = −0.80,	B = 0.91,	B = 1.33,	B = 0.17,	B = 0.25,
	s.e. = 1.23,	s.e. = 1.34,	s.e. = 0.35,	s.e. = 0.34,	s.e. = 0.17,	s.e. = 0.19,
	*p* = 0.66	*p* = 0.57	*p* < 0.001 ***	*p* < 0.001 ***	*p* = 0.30	*p* = 0.18
Interpersonal Relations Behaviors	B = −0.66,	B = −1.44,	B = 0.11,	B = 0.47,	B = −0.06,	B = 0.02,
	s.e. = 1.33,	s.e. = 1.52,	s.e. = 0.39,	s.e. = 0.37,	s.e. = 0.18,	s.e. = 0.15,
	*p* = 0.62	*p* = 0.34	*p* = 0.78	*p* = 0.20	*p* = 0.74	*p* = 0.87
Stress Management Behaviors	B = −0.93,	B = −1.84,	B = 0.67,	B = 1.11,	B = 0.13,	B = 0.14,
	s.e. = 1.38,	s.e. = 1.70,	s.e. = 0.40,	s.e. = 0.39,	s.e. = 0.19,	s.e. = 0.16,
	*p* = 0.50	*p* = 0.28	*p* = 0.09+	*p* = 0.004 **	*p* = 0.50	*p* = 0.40

Note: Health responsibility behaviors, physical activity behaviors, nutrition behaviors, spiritual growth behaviors, interpersonal relations behaviors, and stress management behaviors are subscales of the HPLP-II scale. The HPLP-II summary score is an average of these subscales. The HPLP-II scale ranges from 1 to 4 and is coded such that higher scores denote more frequent health-promoting behaviors. Linear mixed effects models were conducted for 12-month weight loss and engagement, and logistic regressions were conducted for 12 month retention. *** *p* < 0.001, ** *p* < 0.01, * *p* < 0.05.

## Data Availability

Restrictions apply to the availability of these data. Data were obtained from Noom and are available by request from the corresponding author with the permission of Noom.
